# Disrupted visual attention relates to cognitive development in infants with Neurofibromatosis Type 1

**DOI:** 10.1186/s11689-025-09599-4

**Published:** 2025-03-14

**Authors:** Jannath Begum-Ali, Luke Mason, Tony Charman, Mark H. Johnson, Jonathan Green, Shruti Garg, Emily J. H. Jones, and the STAARS and EDEN Teams, Mary Agyapong, Tessel Bazelmans, Leila Dafner, Mutluhan Ersoy, Teodora Gliga, Amy Goodwin, Rianne Haartsen, Hanna Halkola, Alexandra Hendry, Rebecca Holman, Sarah Kalwarowsky, Anna Kolesnik, Sarah Lloyd-Fox, Nisha Narvekar, Laura Pirazzoli, Chloë Taylor, Grace Vassallo, Emma Burkitt-Wright, Judith Eelloo, D Gareth Evans, Siobhan West, Eileen Hupton, Lauren Lewis, Louise Robinson, Angus Dobbie, Ruth Drimer, Saghira Malik Sharif, Rachel Jones, Susan Musson, Catherine Prem, Miranda Splitt, Karen Horridge, Diana Baralle, Carolyn Redman, Helen Tomkins

**Affiliations:** 1 Centre for Brain and Cognitive Development, School of Psychological Sciences, Faculty of Science, Henry Wellcome Building, Birkbeck, University of London, Malet Street, London, UK; 2 Department of Forensic and Neurodevelopmental Sciences, Institute of Psychiatry, Psychology and Neuroscience, King’S College London, London, UK; 3 Department of Psychology, Institute of Psychiatry, Psychology and Neuroscience, King’S College London, London, UK; 4 Department of Psychology, University of Cambridge, Cambridge, UK; 5 Division of Neuroscience and Experimental Psychology, University of Manchester, Manchester, UK; 6 Centre for Developmental Neurobiology, Institute of Psychiatry, Psychology and Neuroscience, King’S College London, London, UK; 7 Department of Child and Adolescent Psychiatry, Institute of Psychiatry, Psychology and Neuroscience, King’S College London, London, UK

**Keywords:** Neurofibromatosis Type 1, Autism spectrum disorder, Attention deficit hyperactivity disorder, Visual attention, Eye tracking, Longitudinal

## Abstract

**Background:**

Neurofibromatosis Type 1 is a genetic condition diagnosed in infancy that substantially increases the likelihood of a child experiencing cognitive and developmental difficulties, including Autism Spectrum Disorder (ASD) and Attention Deficit Hyperactivity Disorder (ADHD). Children with NF1 show clear differences in attention, but whether these differences emerge in early development and how they relate to broader difficulties with cognitive and learning skills is unclear. To address this question requires longitudinal prospective studies from infancy, where the relation between domains of visual attention (including exogenous and endogenous shifting) and cognitive development can be mapped over time.

**Methods:**

We report data from 28 infants with NF1 tested longitudinally at 5, 10 and 14 months compared to cohorts of 29 typical likelihood infants (with no history of NF1 or ASD and/or ADHD), and 123 infants with a family history of ASD and/or ADHD. We used an eyetracking battery to measure both exogenous and endogenous control of visual attention.

**Results:**

Infants with NF1 demonstrated intact social orienting, but slower development of endogenous visual foraging. This slower development presented as prolonged engagement with a salient stimulus in a static display relative to typically developing infants. In terms of exogenous attention shifting, NF1 infants showed faster saccadic reaction times than typical likelihood infants. However, the NF1 group demonstrated a slower developmental improvement from 5 to 14 months of age. Individual differences in foraging and saccade times were concurrently related to visual reception abilities within the full infant cohort (NF1, typical likelihood and those with a family history of ASD/ADHD).

**Conclusions:**

Our results provide preliminary evidence that alterations in saccadic reaction time and visual foraging may contribute to learning difficulties in infants with NF1.

**Supplementary Information:**

The online version contains supplementary material available at 10.1186/s11689-025-09599-4.

## Introduction

Neurodevelopmental disorders affect up to 10% of children in the UK and can substantially affect quality of life [51]. The majority of conditions are associated with genetic changes present from conception [69], and there are thus likely to be changes in neurocognitive development from infancy. Understanding the infant neurocognitive changes that precede the emergence of later behavioural symptoms is critical to uncovering causal pathways to these conditions [48]. However, since many conditions are not diagnosed until patterns of behavioural difficulties become clear in childhood, our understanding of changes in early neurocognitive development has historically been limited. To this end, researchers have turned to prospective longitudinal studies of infants who have a higher likelihood of developing a neurodevelopmental condition. The majority of such studies use a familial design, where infants with a first degree relative with a condition like ASD or ADHD have an approximately 10–20% chance of developing ASD [12, 67] or ADHD themselves [61, 14, 15] and are also more likely to experience related cognitive or language delays [57]. These infants are followed from early infancy to early childhood, where developmental outcome can be ascertained [45]. Such studies have revealed early changes in both brain and behaviour that may predict both symptoms of ASD and ADHD [4, 34, 36, 60, 62] and associated cognitive or language difficulties [13, 49]. However, one limitation of such studies is that insights may be restricted to infants with a familial route to neurodevelopmental conditions. Whilst common genetic variation is important in pathways to autism, a substantial proportion of autistic people (currently estimated at 20–40%) also show more penetrant de novo genetic changes [92]. Thus, more recently, familial designs have been complemented with prospective studies of infants with genetic conditions known to raise the likelihood of neurodevelopmental conditions.

One example of a monogenetic condition associated with increased likelihood for ASD and/or ADHD is Neurofibromatosis Type 1 (NF1). NF1 is a common autosomal dominant condition that affects up to 1 in 2700 children [27]. The disorder is caused by a mutation on the NF1 gene on chromosome 17q11.2, which is important in intracellular signalling, learning and synaptic plasticity [21]. Approximately 50% of cases are inherited and 50% arise de novo [16]. Although most widely known for its cutaneous manifestations, the majority of difficulties experienced by preschool children with NF1 are cognitive, social and behavioural [42]. Children with NF1 have an elevated likelihood of ASD, with prevalence rates between 10–30% Chisholm et al (2018) relative to the 1% in the general population. An additional 30% of children with NF1 show subclinical ASD traits at a mild to moderate severity [64, 70], and between 30–50% of children fulfil the diagnostic criteria for ADHD [30, 42, 58, 73]. Cognitive impairments are common [22], cause significant difficulties at school [30, 42] and have a significant impact on quality of life [91]. For example, one recent study of 206 children with NF1 noted that 80% showed significantly lower scores in at least one cognitive, behavioural or academic domain [32]. These cognitive or learning difficulties are not necessarily related to IQ. Within people with NF1, IQ is normally distributed around a mean of 90; though broader deletions that extend beyond the NF1 gene may be associated with lower IQ. Thus, intellectual disability (defined as an IQ < 70) is relatively rare [65]. Notably, children with a family history of NF1 may experience greater difficulties than those without [32, 41]. Thus, prospective longitudinal studies of infants with NF1 can complement studies of infants with a family history of autism and ADHD and allow us to study the early neurocognitive predictors of both neurodevelopmental traits and cognitive differences. Further, given the genetic cause of NF1 in children is well understood, studying this population holds the potential for a deeper understanding of the neurobiological mechanisms underpinning the neurocognitive changes observed.

One domain of interest is that of visual attention. A recent meta-analysis suggests a Hedges g effect size of around −0.52 for differences in attention in NF1, and visual attention is likely to be particularly relevant given the prevalence of visuospatial differences (effect size −0.85) [22]. Visual attention is also an important modality through which young infants explore their environment, particularly when their early motor skills limit more active exploration. Attention can be measured from very early infancy using either behavioral coding or eyetracking approaches, and is thus a tractable domain for exploring developmental trajectories in NF1. Indeed, several previous studies have identified alterations in visual attention in children with NF1. For example, Lewis et al. [55] used eye tracking methods to examine looking behaviour to faces in complex scenes, in ten-year-old children, those with NF1 looked less at faces in naturalistic scenes as compared to typically developing children. Using a visual habituation paradigm Hocking et al. [39] found that 2–5 year olds with NF1 habituated more slowly to repeated stimuli when compared to both typically developing children and those with a diagnosis of ASD. Interestingly, the NF1 group demonstrated greater levels of attention to repeated stimuli at the expense of the novel stimuli. Slower habituation also associated with increased ASD symptomatology, consistent with evidence that young children with ASD also show prolonged habituation times, particularly to faces [90].

Although visual attention differences have been investigated in children with NF1, it is presently unclear how early in development these changes emerge. Within the (typical) developmental literature, it has been well established that visuospatial coordination of attention is one of the earliest-emerging volitional infant behaviours [10] and thus early-emerging difficulties could potentially have cascading effects on later visuospatial cognition. Eyetracking is a technique that enables the precise measurement of visual attention in infancy through the use of infrared light to detect the direction of gaze. A long history of infant eyetracking and gaze coding research has shown that individual differences in infant attention can relate to later cognition [18, 79, 80], and ASD and ADHD-related traits [25, 52, 60]. Models of infant visual attention distinguish between exogenous orienting (shifting from one spatial location to another in response to an external cue) and endogenous control (an internally-driven shift from one location to another; [19]).

In infancy, exogenous attention shifting is often measured using the Gap-Overlap task, in which the infant fixates on a central stimulus and then is attracted to shift their gaze to the sudden appearance of a peripheral stimulus under competition and non-competition conditions [26, 43]. Endogenous control can be measured during free viewing of naturalistic scenes containing salient (usually social) and less salient features [88, 89]. Typically, patterns of attention shift over the first years of life from being controlled primarily by reflexive salience-driven mechanisms to a more controlled, experience dependent process with a greater reliance on cortical control [45, 46, 83]. Thus, typical developmental changes in visual attention may include faster orienting, increased/faster disengagement to competing stimuli and decreased attention to (socially) salient stimuli as infants age [17, 29].

Previous longitudinal studies from infancy (in other populations) have indicated that individual differences in measures of visual attention can predict later developmental outcomes. For example, in *early* infancy, shorter fixation durations during static viewing have been related to both later ASD [88, 89] and ADHD in later childhood [68]. A long history of research in typically developing infants has linked ‘short looking’ during presentation of repeated stimuli and faster processing speed with later increased IQ [72]. In *later* infancy, slower orienting times on visual attention shifting measures and slower change with development have been related to later ASD in toddlerhood [24–26]. Further, slower developmental decreases in looking to a salient stimulus in a visual array between 10 and 14 months have been related to reduced executive functioning [38] and increased ADHD symptoms [36] in later development. As infants grow older, more rapid exploration (or foraging) of a visual scene is typical, with ‘sticky fixations’ on salient areas considered immature and a hallmark of an inability to disengage from a stimulus [19, 40].

In summary, the development of exogenous and endogenous attention place an important constraint on how infants forage for information about the world. Given widespread reports of attention alterations in children with NF1, understanding whether these differences are present from very early in infancy is important to determining whether they could be a target for early intervention. This may be feasible, because eyetracking is a relatively low cost and scalable measure that can be used for gaze contingent attention training in early development (e.g., [34, 71]. Further, identifying whether early changes in visual attention relate to later broader measures of cognitive development is important as a first step towards determining whether visual attention may lie on the developmental path between the effects of NF1 deletion on the brain and real-world impacts on learning outcomes.

In the present study, we first examined exogenous shifting and endogenous attention control in a group of infants with NF1 compared to typically developing infants. To examine the specificity of any differences to NF1, we investigated the same questions within a cohort that had a familial history of ASD and/or ADHD and thus, an elevated likelihood of developing ASD and/or ADHD and related developmental difficulties. Infants were assessed longitudinally at 5, 10 and 14 months on an eyetracking battery and a range of other behavioural measures [31]. In the present analysis, we used saccadic reaction times from the Gap-Overlap task as a measure of exogenous visuospatial attention shifting, and measures of looking time and direction to a static visual array to measure deployment of endogenous attention towards and away from a salient stimulus (here a face). We measured initial orienting to the face within an object array presentation (included in an array with four other distractor stimuli), and subsequent duration of face looking during the remainder of the 10 s slide duration. Faster attention shifting has been linked (in typically developing cohorts) to higher IQ [79]. Given that NF1 populations tend to have lower IQs, we predicted that exogenous visuospatial attention shifting would be slower in NF1 infants relative to typically developing infants. We also expected the NF1 group to be slower relative to the family history groups. Previous research has shown that, in early infancy, shorter fixation durations during static viewing has been linked to later ASD and ADHD [88, 68] and thus, we would expect attention shifting to also be faster in this cohort relative to our NF1 infants.

In infant populations with a family history of ASD/ADHD, it has been found that slower developmental improvements in the speed of shifting from a salient visual stimulus has been linked to poor effortful control [38]. Given children with NF1 often have poorer executive functioning/effortful control [2], we hypothesised that our NF1 sample would also show slower disengagement from the salient stimulus (i.e., *more* face looking).

Previous evidence has shown that differences emerge over the first year in infants with later ASD or executive functioning difficulties [25, 38]. As such, we expected that group differences in visual attention would increase over developmental time. We also ask whether these visual attention differences are related to visual cognition abilities (specifically the visual reception subscale of the Mullen Scales of Early Learning,Mullen, 1995) and whether the pattern of effects is consistent with the broader difficulties in cognition observed later in development in NF1.

## Methods

### Participants and procedure

28 infants with a clinical diagnosis of Neurofibromatosis Type 1 (NF1) were recruited into a longitudinal study running from 2013 to 2019 (EDEN: [31]). The comparison cohort was a group of *n* = 161 infants with a first degree relative with ASD (FH-ASD) and/or ADHD (FH-ADHD, FH ASD + ADHD) and infants who had no first degree relatives with a diagnosis of ASD and/or ADHD (Typical Likelihood,TL) who were recruited as part of the STAARS study [5]. In both cohorts, infants were tested longitudinally at 5, 10 and 14 months (see SM1 for full recruitment and categorisation processes).

We first compared our cohort with NF1 to infants who had no first-degree relatives with a diagnosis of ASD or ADHD, and for whom parents reported no developmental concerns (Typical Likelihood group; TL). Secondly, we compared the NF1 group to infants with first degree relatives with ASD and/or ADHD (FH-ASD, FH-ADHD or FH-ASD + ADHD). The TL group was largely recruited from a volunteer database at the Centre for Brain and Cognitive Development, Birkbeck University of London. At the time of enrolment, none of the infants in this cohort had a known medical or developmental condition. Participants in the NF1 cohort were recruited through local medical and genetic centres. All participants had their diagnosis confirmed via molecular testing of cord blood samples or clinical diagnosis based on NIH consensus criteria [85] and had no other developmental concerns at the time of the visits. Infants with an older sibling/parent with ASD and/or ADHD were recruited through online advertising, word of mouth, magazines/media, and clinical referrals. Inclusion criteria for all groups included full-term birth (gestational age greater than 36 weeks).

We examined infant visual attention and its associations with concurrent cognitive function at 5, 10 and 14 months of age. At these timepoints, participants came in for a day long visit and took part in a battery of tasks, including measures of eye tracking (see SM2 for full details). Following the eyetracking tasks, the behavioural measures (e.g., the Mullen Scales of Early Learning; Mullen, 1995) were completed. Infants also took part in EEG, physiological and play based assessments though these are not reported in the current paper (see [3]).

Informed written consent was provided by the parent(s) prior to the commencement of the study. The testing only took place if the infants were in a content and alert state. Ethical approval was granted by the National Research Ethics Service and the Research Ethics Committee of the Department of Psychological Sciences, Birkbeck, University of London. Participant families were reimbursed expenses for travel, subsistence and overnight stay if required. Infants were given a certificate and t-shirt after each visit.

**Fig. 1:**
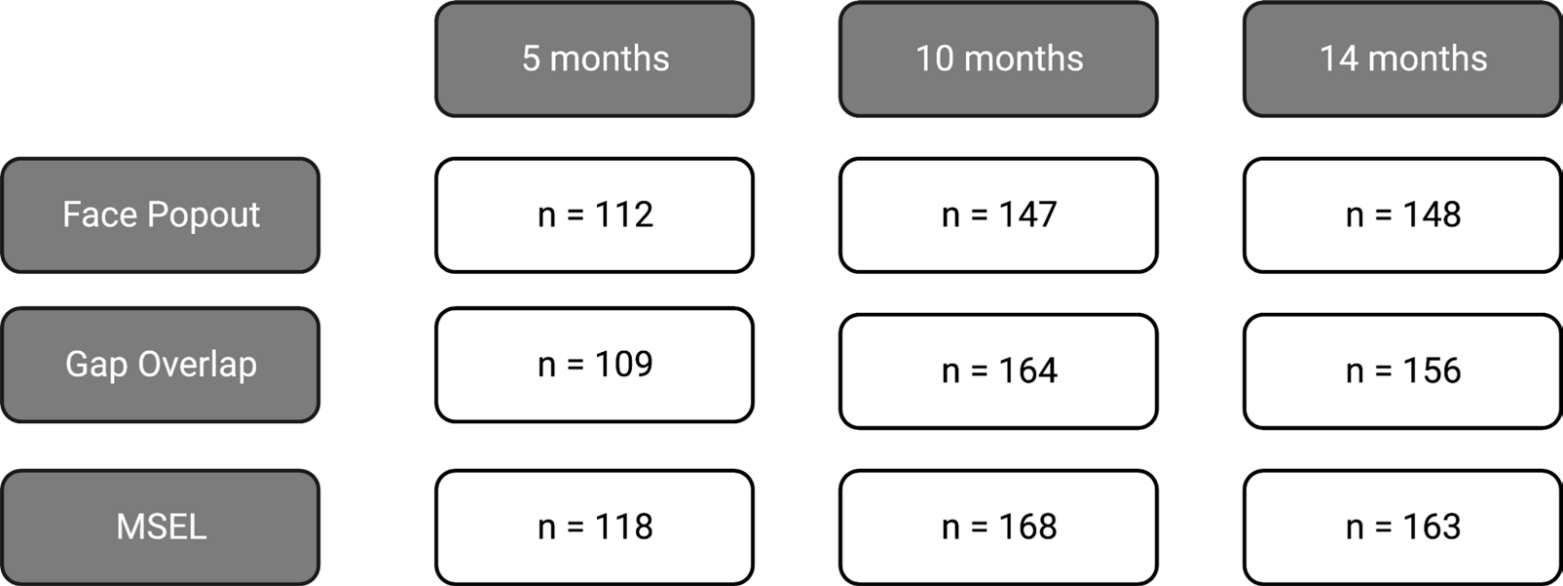
Consort diagram of data availability across measures and timepoint

Data availability varied by timepoint; Fig. 1 gives the number of children providing valid data per timepoint and key dependent measure.

### Measures

#### Eyetracking tasks

Visual stimuli were presented, and eye tracking data acquired, using a Tobii TX-300 eye tracker (see SM 2.1).

*Exogenous attention: Gap-Overlap:* shifts of visual attention were measured using the gap-overlap task [25] which measures the time taken to plan and execute a saccade from a centrally-presented stimulus (CS), to a peripheral stimulus (PS) presented pseudo-randomly at one side of the screen, at distance of ~ 20° of visual angle (at a viewing distance of 60 cm). Each trial started with the onset of a central stimulus (CS), a cartoon image of an analogue clock accompanied by an alerting sound. After a 200 ms period had elapsed, the peripheral stimulus (PS) was presented. In the baseline condition the CS was removed from the screen when the PS was presented. In the overlap condition the CS continued to be presented for the duration of the rest of the trial. In the gap condition the CS was removed from the screen and the PS was presented after a short gap. The PS was a cartoon cloud that appeared on either the left or the right side of the screen and was accompanied by a sound, 3 cm (2.86°) from the edge, rotating at 500° per second until fixated by the participant. A reward stimulus (a star, a sun, a dog, cat, pig, tiger or tortoise which were animated and accompanied by a sound) was then presented at the location of the PS for 1000 ms. The mean saccadic reaction time (SRT) was calculated for the Baseline, Gap and Overlap conditions and logged for analysis to reduce skew,see SM 2.2 for full details.

**Fig. 2:**
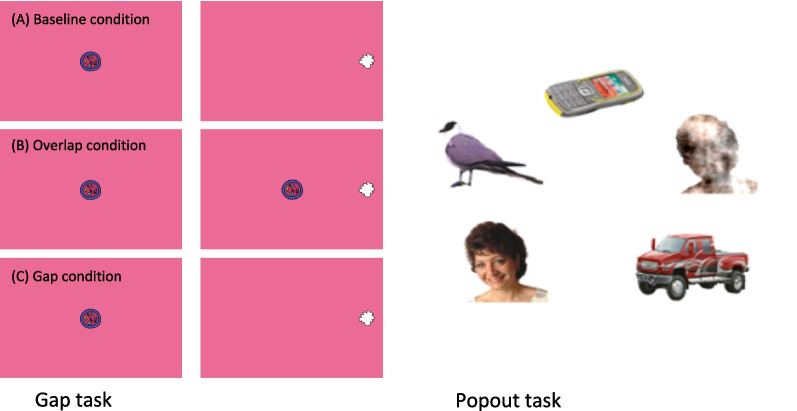
Illustration of the two key variables used in the current study

*Endogenous attention: Face Popout.* Infants were presented with a series of six annular visual arrays (10 s duration) each composed of five objects in different locations on the screen [33, 38]. Each array contained: 1) a face with direct gaze,2) a visual ‘noise’ image generated from the same face presented within the array by randomising the phase spectra of the face whilst keeping the amplitude and colour spectra constant to act as a control for the low-level visual properties of the face stimuli [37]; 3) a bird; 4) a car; and, 5) a mobile phone (see Fig. 2). Each array was presented for 10 s and counter-balanced for the location of the face in the array. Gaze was averaged across eyes, assigned to an area of interest (AOI) and interpolated (< 200 ms missing data). Key dependent variables are the proportion of trials on which the infants first look to the face divided by the number of trials with a valid initial look (reflective of social orienting, “First Look”), and percent looking to faces (reflective of sustained attention to a salient stimulus [26]; see SM 2.3 for full details.

*Mullen Scales of Early Learning (MSEL):* The MSEL (Mullen, 1995) was administered at all time points by trained researchers in the STAARS team. To allow for the greatest level of replicability and consistency across examiners, we have strict guidelines about how Mullens should be administered and marked (see SM3 for further details). These strict administration and scoring guidelines (although those recommended in the Mullen manual) may not be those applied more broadly in the field, and thus may account for relatively poorer performance in this cohort at infant timepoints relative to US norms. The variable of interest for analyses was the Visual Reception subscale (T score), which examines visual and visuospatial processing abilities. Tasks on the Visual Reception scale (commensurate with the ages under investigation) included tracking images of schematic faces, finding hidden objects (object permanence), attention switching and attending to pictures.

#### Data analysis plan

Multilevel growth modelling was used to estimate change in visual attention measures across time using the ‘nlme’ package. For outcomes measured at three time points (5, 10, 14 months), the overall growth trajectory was modelled linearly with fixed effects of all predictors (group, age) on all time terms and a random effect of each participant on intercept and the linear time term. All dependent variables were standardised with respect to the full cohort before modelling. Models were fit using REML to account for missing data. The Typical Likelihood (TL) group were treated as a reference group and parameters were estimated for the NF1 group. Akaike information criteria (AIC) and Bayes information criteria (BIC) were used to examine model goodness of fit, with lower values indicating better model fit.

Analysis 1: Infants with NF1 were compared to Typical Likelihood infants, following previous work [3, 31]. Models were of the form: m1 < -lme(eyetracking score ~ Ageindays * group + sex, random = ~ Ageindays| ID). This allows slopes and intercepts to vary randomly between individuals. If the model failed to converge, the random slope was removed. Sex was removed if it did not show significant effects. For the Gap task, given there are three experimental conditions that produce the same dependent variable the model was of the form m1 < -lme(reaction times ~ Ageindays * label + sex, random = list(~ 1| condition, ~ Ageindays| ID)).

Analysis 2: For eyetracking variables where the NF1 group differed from the Typical Likelihood group, we then compared the NF1 group to the group of infants with a family history of ASD/ADHD (FH-ASD, FH-ADHD, FH ASD + ADHD) using the same model structure to determine whether mechanisms may be shared or distinct.

Analysis 3: For the same set of variables, we examined the relation to concurrent cognitive function in the all groups (NF1, TL, FH-ASD, FH-ADHD, FH-ASD + ADHD). To do this, we used models of the following form: m0 < -lme(VR_TSCR ~ Ageindays*eyetracking score*group, random = ~ 1| ID).

## Results

Table 1 presents clinical and demographic data for infants included in the sample.

**Table 1:** Clinical and demographic data for infants included in the sample

**5 months**					
	**LL**	**NF1**	**ASD**	**ADHD**	**ASD + ADHD**
**n**	26	12	51	15	14
**Sex**	17m, 9f	6m, 6f	26m, 25f	8m, 7f	8m, 6f
**Age in days**	179.19 (14)	192.17 (18.11)	175.27 (20.72)	170 (13.29)	179 (15.16)
**MSEL Early Learning Composite**	85 (9.32)	67 (9.9)	82.98 (10.97)	98.92 (14.64)	85.64 (11)
**10 months**					
	**LL**	**NF1**	**ASD**	**ADHD**	**ASD + ADHD**
**n**	27	19	75	26	21
**Sex**	16m, 11f	9m, 10f	38m, 37f	15m, 11f	12m, 9f
**Age in days**	321.93 (16.7)	327 (17.52)	318.92 (14.43)	324.12 (27.75)	320.81 (15.16)
**MSEL Visual reception**	48.85 (7.99)	42.37 (7.46)	49.77 (9.45)	47.04 (9.8)	48.19 (7.5)
**MSEL Early Learning Composite**	88.89 (12.19)	79.11 (10.34)	87.88 (15.14)	85.04 (15.61)	85.57 (16.42)
**14 months**					
	**LL**	**NF1**	**ASD**	**ADHD**	**ASD + ADHD**
**n**	23	24	70 72	24	20
**Sex**	13m, 10f	10m, 14f	38m, 34f	17m, 7f	12, 8f
**Age in days**	445.83 (18.29)	450.37 (24.38)	451.01 (18.19)	450.58 (21.89)	448.55 (20.53)
**MSEL Visual reception**	35.09 (8.88)	33.33 (5.3)	37.6 (8.7)	36.33 (5.82)	33.8 (6.5)
**MSEL Early Learning Composite**	78.78 (11.99)	72.87 (7.21)	78.04 (11.87)	79.08 (21.89)	73.55 (14.84)

Comparison of precision, accuracy and measures of data quantity indicate that data quality was equivalent in the two groups in Analysis 1 (SM4.1) for the accuracy and data quantity metrics. Since precision values improved with age (lower values indicate less variability) and were significantly lower in the NF1 group, this variable was included as a covariate in sensitivity analyses (see SM4.3; the pattern of results was substantively the same as that reported in the main text).

### Analysis 1: Infants with NF1 compared to infants with a Typical Likelihood (TL)

#### Exogenous shifting

**Fig. 3:**
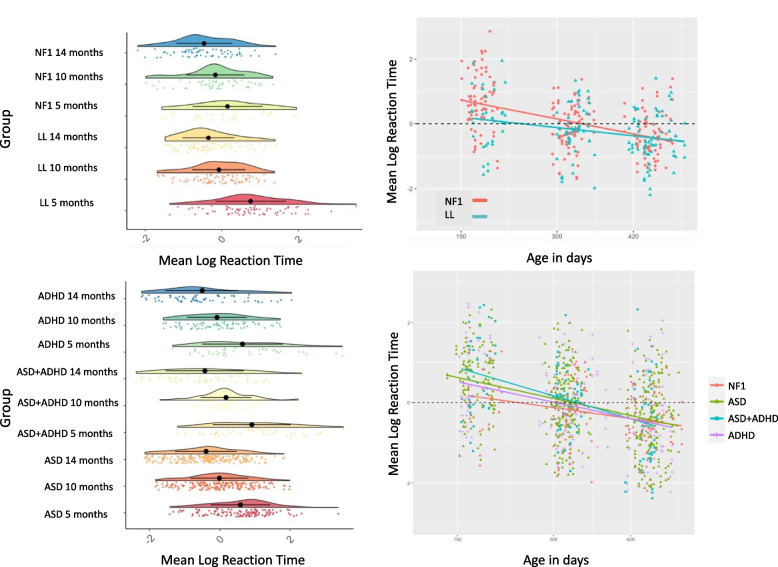
Exogenous attention shifting measured using the gap-overlap task in the NF1 and TD groups. Left: Changes in reaction time with age at a group level; Right: Trajectories of change in reaction times by age in days

Gap reaction times: If Condition was included as a fixed effect with the Baseline as the reference category (lme(reaction times ~ Age * group*condition + sex, random = list(~ Ageindays| ID); AIC = 863.31, BIC = 929.33, loglik = −414.65) it did not significantly interact with Group (Gap effect: t = −0.83, *p* = 0.41; Overlap effect t = 1.07, *p* = 0.29) and given the higher BIC (compared to AIC) it was instead included as a random effect. Sex was retained in the model as it was a significant predictor of reaction times, such that males showed faster reaction times than females [t(166) = −2.62, *p* = 0.01; CI = −0.77 to −0.07; mean RT 5.72 vs 5.66 respectively]. Thus, the final model was of the form: reaction times ~ Age * group + sex, random = list(~ 1| condition, ~ Age| ID; AIC = 872.67, BIC = 911.75, loglik = −426.33). Reaction times got faster with age [t(200) = 7.81, *p* < 0.001; CI = −0.005 to −0.003]. The NF1 group showed faster reaction times than the TL group [t(166) = −2.75, *p* = 0.01; CI = −1.32 to −0.22) and a slower developmental improvement [t(200) = 2.24, *p* = 0.02; CI = 0.0001 to 0.0003); see Table 2 and Fig. 3.

**Table 2:** Eye tracking key variables mean (SD)

5 months					
	**LL**	**NF1**	**ASD**	**ADHD**	**ASD + ADHD**
**Gap Baseline RT (log)**	5.75 (.12)	5.67 (.12)	5.75 (.16)	5.78 (.2)	5.76 (.14)
**Gap Gap RT (log)**	5.61 (.12)	5.5 (.09)	5.57 (.1)	5.58 (.14)	5.59 (.17)
**Gap Overlap RT (log)**	6.09 (.21)	6.02 (.18)	6.09 (.25)	6.04 (.24)	6.16 (.28)
**Popout Face Proportion Looking**	.48 (.16)	.5 (.18)	.48 (.16)	.49 (.19)	.44 (.15)
**Popout Looking to Face first proportion**	.52 (.24)	.42 (.2)	.39 (.23)	.39 (.19)	.4 (.24)
**Accuracy Degree**	2.09 (.54)	1.83 (.55)	2.13 (1)	2.16 (.6)	2.25 (.66)
**Precision Degree**	2.41 (.57)	1.59 (.33)	2.43 (1)	2.45 (.7)	2.57 (.71)
**10 months**					
	**LL**	**NF1**	**ASD**	**ADHD**	**ASD + ADHD**
**Gap Baseline RT (log)**	5.71 (.11)	5.68 (.11)	5.73 (.15)	5.69 (.14)	5.73 (.14)
**Gap Gap RT (log)**	5.47 (.08)	5.44 (.09)	5.46 (.11)	5.45 (.09)	5.48 (.09)
**Gap Overlap RT (log)**	5.87 (.16)	5.91 (.19)	5.93 (.22)	5.92 (.21)	5.98 (.14)
**Popout Proportion Looking**	.45 (.13)	.49 (.11)	.5 (.16)	.47 (.17)	.39 (.13)
**Popout Looking to Face first proportion**	.59 (.23)	.55 (.3)	.59 (.24)	.56 (.25)	.47 (.26)
**Accuracy Degree**	1.59 (.41)	1.68 (.57)	1.75 (.54)	1.89 (.42)	1.96 (.75)
**Precision Degree**	1.89 (.46)	1.53 (.4)	2.04 (.59)	2.2 (.44)	2.27 (.85)
**14 months**					
	**LL**	**NF1**	**ASD**	**ADHD**	**ASD + ADHD**
**Gap Baseline RT (log)**	5.65 (.11)	5.64 (.12)	5.65 (.13)	5.61 (.15)	5.65 (.18)
**Gap Gap RT. (log)**	5.42 (.08)	5.39 (.09)	5.41 (.12)	5.38 (.11)	5.42 (.16)
**Gap Overlap RT (log)**	5.85 (.16)	5.84 (.17)	5.84 (.18)	5.85 (.27)	5.8 (.22)
**Popout Proportion Looking**	.32 (.13)	.46 (.17)	.41 (.13)	.4 (.19)	.38 (.09)
**Popout Looking to Face first proportion**	.51 (.26)	.64 (.21)	.62 (.22)	.5 (.25)	.57 (.22)
**Accuracy Degree**	1.61 (.46)	1.5 (.28)	1.73 (.47)	1.89 (.53)	1.91 (1.01)
**Precision Degree**	1.9 (.5)	1.47 (.31)	2.02 (.5)	2.2 (.52)	2.22 (1.24)

N.B: Precision measures the degree of variability. Accuracy measures the difference between a gaze point and where it should be on the screen. As such, smaller values for both metrics indicate better precision and accuracy

### Endogenous attention

#### First looks to face

**Fig. 4:**
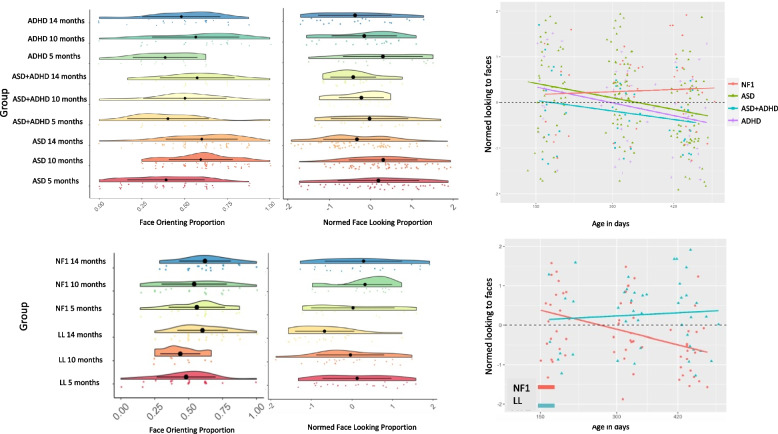
Endogenous attention measured using the Popout task in the NF1 and TD groups. Left: Changes in face orienting and proportion looking to face with age at a group level; Right: Trajectories of change in proportion looking to faces by age in days

The final model was of the form: proportion face orienting ~ Age * group + sex, random = list(~ Age| ID). There was a marginally significant Group difference in the proportion of trials on which infants oriented to the face before other stimuli such that infants with NF1 oriented slightly less often [t(52) = −1.9, *p* = 0.06; CI = −5.86 to 0.01] and a marginally significant interaction with Age such that infants with NF1 improved slightly more with Age [t(65) = 1.94, *p* = 0.06; CI = −2.22 to 0.00]. There was no main effect of Age [t(65) = 0.009, *p* = 0.99; CI = −5.09 to 0] and a marginal effect of Sex such that female infants showed slightly stronger face orienting than male infants [t(65) = 1.94, *p* = 0.06; CI = −3.53 to 0.18]. As a group, infants robustly looked at the face more than chance [0.42 vs 0.2 chance, t(65) = 3.99, *p* < 0.001; Fig. 4].

#### Proportion looking to face

The effect of Sex was not significant [t(51) = 1.56, *p* = 0.12, CI = −0.07 to 0.58], so was not included in the final model. The final model took the form: proportion of face looking ~ Ageindays * group, random = ~ Ageindays| ID (AIC 365.18; BIC 387.34; log likelihood −174.6). Looking to the Face decreased with Age [t(52) = −2.76, *p* = 0.008; CI = −0.006 to −0.001] but decreased more slowly with Age in the NF1 group [t(52) = 2.08, *p* = 0.04; CI = 0.0 to 0.01; Fig. 4). There was no overall Group difference [t(52) = −1.25, *p* = 0.22; CI = −2.17 to 0.5].

#### Analysis 2: Comparison to infants with a family history of autism and/or ADHD

In order to determine the specificity of the effects found in the previous analyses, we compared our NF1 group to those with a family history of ASD and/or ADHD (FH-ASD, FH-ADHD, FH-ASD + ADHD).

#### Exogenous shifting

Reaction times: Sex was not a significant predictor of saccadic reaction times [t(464) = 0.24, *p* = 0.81; CI = −0.12 to 0.16] and was not included in the final model. Thus, the final model was of the form: saccadic reaction times ~ Ageindays * group, random = list(~ 1| condition, ~ Ageindays| ID); AIC = 2577.8, BIC = 2641.7, loglik = −1275.91). Saccadic reaction times got faster with Age [t(546) = −3.42, *p* < 0.001; CI = −0.004 to −0.001). The NF1 group showed faster saccadic reaction times than all other groups [FH-ASD: t(465) = 2.42, *p* = 0.02, CI = 0.12 to 1.18; FH-ASD + ADHD t(465) = 3.66, *p* = 0.003, CI = 0.56 to 1.85; FH-ADHD t(465) = 2.81, *p* = 0.005, CI = 0.27 to 1.52] and a slower developmental improvement than other groups with a family history of ADHD [FH-ASD + ADHD: t(545) = −2.89, *p* = 0.004, CI = −0.004 to −0.001; FH-ADHD: t(546) = −2.53, *p* = 0.01, CI = −0.004 to −0.001], but not those with a family history of ASD only [FH-ASD: t(546) = −1.77, *p* = 0.08, CI = −0.003 to 0].

#### Endogenous attention: proportion looking to Face

Sex was a significant predictor such that female infants looked more at faces than male infants [t(144) = 2.09, *p* = 0.04, CI = 0.01 to 0.43) and thus was included in the final model (proportion of face looking ~ Age * group + sex, random = ~ Age| ID; AIC = 824.32, BIC = 872.20, log likelihood = −399.16). The NF1 group did not differ from other groups [ASD: t(144) = 1.13, *p* = 0.26, CI = −0.48 to 1.77; FH-ASD + ADHD: t(144) = 0.30, *p* = 0.77, CI = −1.13 to 1.53; FH-ADHD: t(144) = 0.86, *p* = 0.39, CI = −0.74 to 1.89) and did not show different developmental change to the other groups [ASD: t(150) = −1.68, *p* = 0.09, CI = −0.01 to 0; FH-ASD + ADHD t(150) = −1.08, *p* = 0.28, CI = −0.01 to 0; FH-ADHD t(150) = −1.47, *p* = 0.14, CI = −0.01 to 0]. Thus, the proportion of looking to faces in NF1 were similar to infants with a familial history of ASD and/or ADHD.

#### Analysis 3: Relation to visual reception

Here, for the same eye tracking variables, we examined the relation to concurrent cognitive function across all groups (NF1, TL, FH-ASD, FH-ADHD and FH-ASD + ADHD).

#### Exogenous shifting

The final model was of the form: visual reception ~ Age*reaction time*group + sex, random = list(~ 1| condition, ~ Age| ID); AIC = 9015, BIC = 9148; log likelihood = −4481) and included all infants. Visual reception scores decreased with Age [t(666) = −7.87, *p* < 0.001, CI = −8.82 to −0.92) and were lower in the NF1 group [t(550) = −4.65, *p* < 0.001, CI = −2.25 to −9.13] and ADHD groups [t(550) = −2.17, *p* = 0.04, CI = −1.29 to −0.64] particularly at the younger timepoints [Age by NF1: t(666) = 3.19, *p* = 0.002, CI = 1.19 to 0.05; Age by ADHD t(666) = 2.18, *p* = 0.03, CI = 2.02 to 0.04]. Faster reaction times were associated with stronger visual reception overall [t(666) = −2.42, *p* = 0.02, CI = −6.17 to −0.04]; this was weaker in the NF1 group [Group by Reaction time interaction: t(196) = 2.57, *p* = 0.01, CI = 1.19 to 0.05] and stronger in younger infants [Age by Reaction time interaction: t(196) = 2.05, *p* = 0.04, CI = 5.75 to 0.02].

#### Endogenous attention: Proportion Looking to Face

The relation between visual attention to faces and cognition was assessed using the following model: visual reception ~ Age*face looking*group + sex, random = ~ Ageindays| ID; AIC = 2708, BIC = 2800, log likelihood = −1330). Here, the interaction term was significant and therefore the differential effects by Group were included. Visual reception scores decreased with Age (t(173) = −2.89, *p* = 0.004, CI = −0.05 to −0.01) and were lower in the NF1 group (t(172) = −2.56, *p* = 0.01, CI = −25.64 to −1.76). More looking time to the face (proportionally) was associated with lower visual reception overall [t(173) = −2.29, *p* = 0.02, CI = −16.62 to −0.89] and the association between visual reception and looking time was stronger in older infants [age by looking interaction: t(173) = 2.45, *p* = 0.01, CI = 0.01 to 0.05). Both overall relations between looking time and visual reception [Group by looking time: t(173) = 3.34, *p* = 0.001, CI = 8.53 to: 35.27] and change with Age [Group by Age: t(173) = −3.18, *p* = 0.002, CI = −0.1 to −0.02) were stronger in infants with NF1.

## Discussion

Differences in attention are common in children with NF1 [22]. Here, we show that differences in visuospatial attention emerge in early infancy and may relate to broader differences with visual cognition. Specifically, we show that infants with NF1 are initially faster to respond to a sudden onset peripheral stimulus, but that reaction times decrease more slowly with age than in typically developing infants. Further, infants with NF1 show slower changes in reaction times than infants with older siblings with ASD or ADHD, who are also vulnerable to a range of developmental difficulties. Faster reaction times associated with stronger visual reception skills in infants with, and without, a family history of autism or ADHD, but this relationship was weaker in infants with NF1. This may suggest that the faster early reaction times in NF1 are not contributing to cognitive development for this cohort. Further, in an endogenous attention task infants with NF1 showed a slower decrease in looking to a salient stimulus with age than typically developing infants. Infants with NF1 showed a similar profile to infants with older siblings with ASD/ADHD, who have been previously shown to show developmental slowing in this task [25, 38]. More looking to the face (reduced disengagement from the salient stimulus associated with lower concurrent cognitive skills across all groups, particularly at older ages,this was strongest in infants with NF1. Taken together, these results suggest that both exogenous visuospatial attentional orienting and endogenous attentional control may be altered in NF1, and the latter may relate to broader aspects of visual cognition.

### Exogenous orienting

The ability to shift attention to a peripheral stimulus is present from birth [56], and at this age is primarily subcortically mediated by structures such as the cerebellum, brain stem and superior colliculus [11, 45]. Over the first six months, saccadic control becomes increasingly cortical, involving structures like the frontal eye fields and prefrontal cortex [7]. Thus, examining basic saccadic reaction time can provide insight into the developing brain. In early life, typically developing infants demonstrate long, sustained fixations on a visual stimulus; “sticky fixations” [19, 40], which reflect an inability to disengage from the visual input. By approximately 4 months of age, infants develop the ability to inhibit these longer fixations [20] and begin to strategically scan a display, encoding and engaging with a wider range of visual information. In terms of exogenous orienting, and the Gap-Overlap task more specifically, this developmental shift may present as initially longer reaction times when shifting from the central to the peripheral stimulus, with disengagement reaction times decreasing as infants’ age.

In the present study, infants with NF1 showed *faster* reaction times than typically developing infants; Fig. 3 shows that this is strongest at the 5-month timepoint. Shorter fixation durations during viewing of static arrays (indicating faster shifting between array elements) at 6 months have been previously related to later attention and cognitive difficulties [68], and to later diagnosis of ASD [65, 66]. Faster reaction times in an attention-shifting task could also indicate diminished engagement with the central stimulus; if infants are not as interested/engaged with the stimulus, they are more likely to be captured by the peripheral stimulus and disengage faster from the original, central stimulus. In future studies, it may be fruitful to use EEG (and particularly the Nc component) to examine the degree of engagement with a stimulus.

In terms of the results of this study, it is possible that our NF1 group demonstrating faster reaction times in the attention shifting task is related to observations of poorer sustained attention in older children with NF1 [44]. For example, Michael et al. [59] found that older children with NF1 showed over reactivity and longer inspection of visual signals that were presented outside the current focus of attention,faster reaction times to a peripheral stimulus in the present cohort may potentially capture similar processes. Interestingly, one previous study reported that higher GABA levels were related to faster reaction times in a behavioural go/no go task in NF1 [75] and a second study reported associations between higher GABA and faster RTs in a visuospatial working memory task [31]. Thus, testing the degree to which our infant findings reflect alterations in cortical inhibition is an important direction for future research. Notably, our findings were not compromised by differences in data quality,although differences in precision (the stability of gaze points around the centre during a fixation) were observed, precision was better in infants with NF1 (as indicated by smaller variability values) and was associated with *longer* reaction times, indicating that this worked against the direction of our results. Indeed, covarying for precision if anything made the findings stronger.

Although saccadic reaction times were faster in infants with NF1 in early development, infants with NF1 showed a slower developmental change in reaction time than typically developing infants. Reduction in saccadic reaction time with age is a robust finding in typically developing infants that has been replicated many times [35]. Thus, the diminished rate of change in speed of saccadic reaction time with age seen in NF1 suggests that the visual orienting system is not maturing typically. Indeed, infants with NF1 also differed from infants with older siblings with ASD and/or ADHD, suggesting that this difference is not generically observed in infants with risk factors for any neurodevelopmental condition. In other cohorts, developmental change has provided a more sensitive predictive measure than static measures taken at single timepoints. For example, [25] showed that slower developmental decreases in attention shifting in competition conditions between 6 and 12 months predicted later diagnosis of ASD in infants with a family history of the condition. Thus, measures of developmental change may provide more sensitive measures of neurocognitive development for use in later prediction.

Within the broader group of infants (i.e., our NF1 group as well as those with/without a family history of ASD/ADHD), faster saccadic reaction times were related to a broader measure of visual cognition, with effects stronger at younger ages. However, this effect was significantly weaker in infants with NF1, suggesting that different mechanisms underlie faster orienting in infants with NF1 as in other infants. The Mullen visual reception scale measures skills such as object permanence, object recognition and attention switching. Thus, the present results suggest that basic control of visual attention is related to this broader suite of visual cognitive abilities in typical development. Other studies have also suggested that visual attention relates to visual cognition later in development [78–80]. Further, subgroups formed from profiles of performance on visual attention tasks in the first year of life can explain variance in later visual short term memory [81]. Relatedly, it is important to consider the potential cascading effects of atypical visual attention in terms of social-communication development. Subtle differences in eye movements may change the way that infants with NF1 interact with their environment. Specifically, faster saccadic reaction times may reflect reduced engagement with stimuli. In social contexts, this could result in fewer successful episodes of joint attention which could have downstream consequences for social development. Future longitudinal modelling could address these possibilities. Integrating eyetracking measures with other modalities may also be important. For example, cortical markers of attention engagement have also been related to later cognitive skills in typically developing infants and infants with a family history of ASD [8, 49], whilst late-stage visual evoked potentials during visual attention have been identified in children with NF1 [74]. Examining similar EEG markers within the present cohort will be an important next step.

### Endogenous orienting

To measure endogenous attention, we presented infants with a static array containing a salient stimulus (a face) and four other comparison objects [33]. Previous work has shown that infants typically orient first to the face, then scan around the array. With development, infants become more efficient at processing the face and moving on, such that looking times to the face typically decrease with developmental time [50]. Previous work has shown that infants with older siblings with ASD show a slower developmental decrease in face looking than typically developing infants, and within this group this reduced developmental decrease is associated with poorer later effortful control at age 3 [38]. Additionally, slower developmental decreases in visual attention appear to be more related to ADHD in later childhood [36]; though importantly this needs further investigation. In the present study, infants with NF1 also showed reduced developmental decrease in looking times with age (Fig. 3) and were significantly different from typically developing infants but not from infants with older siblings with ASD and/or ADHD. When longer-term follow-up data is available, we will examine if ASD and ADHD have different patterns of endogenous visual attention in the group of infants with NF1. Alterations in endogenous attention may also represent precursors of differences in sustained attention observed in children with NF1. For example, pre-schoolers with NF1 had longer look durations and slower habituation to repeating non-social visual stimuli relative to those with idiopathic ASD or typically developing children [32], disengagement from a familiar stimulus may be an area of difficulty in NF1. More broadly, differences in visual attention have been linked to reading difficulties in children with NF1 [86]; exploring whether the phenotypes we observed are related to later reading could provide insights into whether early support could be provided.

### Implications for future work

A clear next step is to examine the predictive validity of the eyetracking measures selected in larger cohorts of infants with NF1 with longer-term follow up data. Such associations may also not be specific to NF1. Population studies looking for early predictors of later cognitive development and studies of infants with other genetic syndromes may benefit from including simple measures of visuospatial attention in their batteries. A further next step would be to examine the translational potential of this work. Animal models of NF1 are available, and have been used to link differences in attention to striatal dopamine [9]; broader cognitive differences are associated with Ras regulation of inhibitory networks [82]. Saccadic responses can be studied in rodents [1], and could be used in animal models of NF1 to test whether similar effects are seen. If so, the neurobiological mechanisms underpinning the effects we see and their links to the neurofibromin gene can be further probed. This may also prove fruitful in the development of new treatments for NF1. Recent trials have tested the efficacy of medications such as statins [6, 54, 84] with encouraging results; it may be that the development of new simple tasks that can be used in both human and animal models will help accelerate the development of new medication options or could be used as future proxy outcome measures. Alternative technology-assisted interventions could also be considered; visual attention training delivered through an eyetracker can produce enhanced changes in saccadic reaction time in typically developing infants and sometimes has generalised effects on infant attention to real world objects [87]. Trialling such attention training programs with infants with NF1 may be a fruitful strategy for future work, though current programs will need augmenting given recent failures to shift attention profiles in infants with parents/older siblings with ADHD [34].

The current study also indicates the potential value for eyetracking to study infants and toddlers with genetic syndromes (e.g., [23, 28, 39, 66, 77]. Such approaches will allow us to move beyond behavioural measures, which may be less sensitive to early developmental differences. Indeed, in the same cohort of infants with NF1 we recently showed very few developmental differences on a range of standardised behavioural measures that assessed cognitive, motor and language skills [31], but we did observe significant differences in EEG measures of habituation that related to autistic traits [3]. Furthermore, others have shown similar differences in EEG in other genetic syndromes (e.g., [53, 76]. Thus, eyetracking and other neurocognitive measures may provide a useful complement to behavioural measures like the Mullen for studies attempting to identify individual differences in infancy. In person assessments like the Mullen require significant training, are lengthy, subjective and may require significant adaptation for cross-cultural use [63]. Indeed, we have previously shown that eyetracking assessments can be more robust and generalisable across sites than subjective behavioural measures in infancy [50]. Neurocognitive measures of visual attention have also proved sensitivity to intervention-related changes in infant cognition [47, 87]. Thus, neurocognitive assessments should be an important part of the assessment protocol in newer population cohorts or global health studies.

### Limitations

Due to the relatively rare nature of NF1, our sample size was relatively small. Data quality was broadly similar in our groups of infants across most measures, though the NF1 infants demonstrated significantly less variability (and thus better) precision. Precision represents the degree of clustering of gaze estimates within a fixation and could reflect either poorer data quality or genuine differences in the stability with which a fixation is maintained. Interestingly, precision was better in the NF1 group than in typically developing infants, which may be related to greater passivity. Notably, results did not change when precision was covaried in analyses, and indeed increased precision was associated with slower reaction times in the gap (the opposite direction to the NF1 effect), suggesting that these differences did not confound interpretations. The similarity in trial numbers, accuracy and percent data obtained between the two groups indicates that eyetracking is generally a feasible method in this special population.

## Conclusion

Young children with genetic syndromes like NF1 experience significant challenges that include cognitive, social and attention problems. These difficulties likely emerge starting from infancy, and yet many children typically only receive help and support for behavioural challenges when their full manifestations become apparent. Studying infant neurocognitive development may help us identify the developmental paths that mediate between genetic risk factors and later symptoms, and in the longer term may help us to develop new early identification or intervention approaches. Here, we show alterations in the very early development of visual attention in infants with NF1; specifically we observed both slower development of endogenous visual foraging and slower developmental changes in exogenously-driven saccadic reaction times. Further, individual differences in foraging and saccade times were concurrently related to broader visual reception skills, suggesting that they may be early mechanistic indicators of individual differences in broader cognitive domains. These simple measures of visual attention hold promise for linking between genes and behaviour because their underpinning neurobiological circuits can be carefully probed. Future work can build on our work to test the potential for interventions targeted towards visual attention, or to use eye tracking techniques in naturalistic settings to examine how screen-based measures of visual attention generalise to real-world contexts.

## Supplementary Information

Additional file 1.

## Data Availability

The datasets generated and/or analysed during the current study are not publically available due to confidentiality constraints within our ethical approvals. However, access may be granted upon completion of a successful Project Affiliation Form via The BASIS/STAARS Network (http://www.basisnetwork.org/) and completion of all requisite data access and sharing protocols. Please get in touch with the corresponding authors to start this process.

## Abbreviations

NF1: Neurofibromatosis Type 1
ASD: Autism Spectrum Disorder
ADHD: Attention Deficit Hyperactivity Disorder
MSEL: Mullen Scales of Early Learning
STAARS: Studying Autism and ADHD in the eaRly yearS
EDEN: Early DEvelopment in Neurofibromatosis type 1
FH-ASD: Family history of ASD
FH-ADHD: Family history of ADHD
FH-ASD + ADHD: Family history of ASD and ADHD
TL: Typical Likelihood
