# Supplementary material for: Disrupted visual attention relates to cognitive development in infants with Neurofibromatosis Type 1
Source: J Neurodev Disord. 2025 Mar 14;17:12. doi: 10.1186/s11689-025-09599-4 (PMC11907931; doi:10.1186/s11689-025-09599-4)
Supplement: Supplementary file 1 — Additional file 1. [file 11689_2025_9599_MOESM1_ESM.docx]

Supplementary Materials: Disrupted visual attention relates to cognitive development in infants with Neurofibromatosis Type 1

Jannath Begum-Ali, Luke Mason, Anna Kolesnik, Mark H. Johnson, Jonathan Green, Shruti Garg & Emily J.H. Jones and the STAARS and EDEN Teams

[1. Participant details 2](#_Toc143100988)

[2. Further details of eye tracking measures 5](#_Toc143100989)

[2.1 Data quality assessment 5](#_Toc143100990)

[2.2 Gap-overlap task 6](#_Toc143100991)

[2.2.1 Stimulus presentation 6](#_Toc143100992)

[2.2.2 Data extraction: 7](#_Toc143100993)

[2.3 Face Pop-out task 7](#_Toc143100994)

[2.3.1 Stimulus presentation: 7](#_Toc143100995)

[2.3.2 Data extraction: 8](#_Toc143100996)

[3. Further details of Mullen Scales of Early Learning 9](#_Toc143100997)

[4. Results 10](#_Toc143100998)

[4.1 SM Analysis 1: Data quality 10](#_Toc143100999)

[4.2 SM Analysis 2: Confirmation of task performance 11](#_Toc143101000)

[4.3: SM Analysis 3: Controlling for precision 14](#_Toc143101001)

# Participant details

Participants were recruited for a longitudinal study (STAARS) running from 2013 to 2019 from a volunteer database, community flyers, internet adverts and clinical networks. Participant families were reimbursed expenses for travel, subsistence and overnight stay if required. Infants were given a certificate and t-shirt after each visit.

Information about diagnostic status was ascertained through a number of methods. Before families enrolled in the study, a telephone screening form was used to determine the presence of ASD and ADHD in family members. During their infant’s visit to the lab, the parent/caregiver also completed a “Medical and Psychiatric History Interview” (Appendix A) with the researcher. The telephone screening form and this formal interview at a study visit were the primary sources of information about diagnostic status. In addition, we asked for medical updates at each study visit and re-administered the Medical and Psychiatric History Interview at the 2-year timepoint. We also requested diagnostic letters and asked parents to complete the DAWBA (Goodman, Ford, Richards, Gatward & Meltzer, 2000) ASD and ADHD sections and these were reviewed by the senior clinician (TC). In addition, parents completed the Conners (Conners, 2008) (for ADHD) and the Social Communication Questionnaire (Rutter, Bailey & Lord, 2003) and Social Responsiveness Scale (Constantino, 2012; for ASD) on the family member with a diagnosis and where possible all other family members. This information is used to characterise our sample rather than for exclusionary purposes since, in the UK, NHS clinical diagnoses follow a gold-standard procedure including collation of information from parents, teachers and from in-person assessment that is beyond the scope of this study and more accurate than simple questionnaire measures.

Up to 30% of children with ASD meet criteria for ADHD when prospectively assessed (Simonoff, Pickles, Charman, Chandler, Loucas & Baird, 2008). In clinical practice, the prevalence of dual diagnosis is in practice much lower (Russell, Rodgers, Ukoumunne & Ford, 2014). Given the nature of the co-occurrence between ASD and ADHD and our longitudinal study, sometimes family members would have a suspected diagnosis of ADHD at study entry that would be confirmed later in the study; on other occasions, a family would enrol on the basis of an ASD diagnosis in an older sibling but by the end of the study, they would report that the same sibling was now undergoing assessment for suspected additional ADHD. Where possible, families who reported suspected ADHD at study entry were screened using a shortened version of the Conners. For siblings (aged less than 6 years), a shortened version of the Conners Early Childhood (Conners, 2008, Conners & Goldstein, 2009) form is used. For siblings (6 years or older), a shortened version of the Conners 3 was used. Thresholds for inclusion were the presence of 6 ADHD traits on either the hyperactivity/impulsivity or inattention scale, and a positive score on the impairment scale. For parents a shortened version of the Conners Adults ADHD Rating Scale (CAARS) was used. Thresholds for inclusion were the presence of 5 ADHD traits on either the hyperactivity/impulsivity or inattention scale as per updated DSM V guidelines (see Table 1 for categorisation of the cohort). In terms of the use impairment scores, we used a reduced version of the Conners EC and Conners 3 for individuals under 18 and the CAARS for individuals aged 18+ years. The Conners EC and Conners 3 included questions regarding impairment, as such we also included these questions in our screening forms. In comparison, the CAARS (adult questionnaire) did not include questions regarding impairment. In order to maintain consistency of measure, we did not adapt the CAARS to add impairment questions. Of note, at initial contact with participants, parents were asked if there were any diagnoses of ADHD in the immediate family or if they had any concerns about ADHD. It is only if parents reported concerns that the screening process took place. This is a very similar categorisation protocol to that adopted by other papers/labs using the prospective longitudinal study model in infants at elevated likelihood of ADHD (see Miller et al., 2020; see Table S1 for the number of screened vs diagnosed participants). Families who screened positive on this instrument were then included as a confirmed case. However, it remains likely that within families with ASD, rates of actual ADHD are higher than those captured by our 1/0 diagnostically-based rating system.

| Table S1: Categorisation of elevated likelihood cohorts | | | |
| --- | --- | --- | --- |
|  | **ASD-L** | **ADHD-L** | **ASD+ADHD-L** |
| Parent reported diagnosis in older sibling | 74 | 7 | 15 |
| Parent reported diagnosis in parent | 3 | 18 | 2 |
| Parent reported diagnosis in both older sibling+parent | 3 | 1 | 2 |
| Screened parent (for ADHD traits) |  | 1 | 0 (+1)* |
| Screened older sibling (for ADHD traits) |  | 4 | 0 (+1)** |

*Sibling diagnosed with ASD, also screened for ADHD traits . **Sibling diagnosed with ASD, parent screened for ADHD traits.

Participants in our NF1 cohort were recruited through local medical and genetic centres. All participants had their diagnosis confirmed via molecular testing of cord blood samples or clinical diagnosis based on NIH consensus criteria (Stumpf et al., 1988) and had no other developmental concerns at the time of the visits.

Informed written consent was provided by the parent(s) prior to the commencement of the study. The testing only took place if the infants were in a content and alert state. Ethical approval was granted by the National Research Ethics Service and the Research Ethics Committee of the Department of Psychological Sciences, Birkbeck, University of London. Participant families were reimbursed expenses for travel, subsistence and overnight stay if required. Infants were given a certificate and t-shirt after each visit.

# Further details of eye tracking measures

## 2.1 Data quality assessment

Eye tracking tasks were administered as part of a larger battery (~30 minutes), using a Tobii TX-300 eye tracker (Tobii AB, Stockholm, Sweden) sampling at 120 Hz. The screen had a diagonal size of 23” (58.4 cm ×28.6 cm, 52.0◦×26.8◦@ 60 cm), a native resolution of 1920 ×1080 pixels and an aspect ratio of 16:9. Stimuli were presented on Apple (Apple Inc., Cupertino, CA, USA) Macbook Pro computers, using our custom-written stimulus presentation framework (Task Engine, sites.google.com/site/taskenginedoc/), running in Matlab R2020b (The MathWorks Inc., Natick, MA, USA) using Psychtoolbox 3.

Before calibration started, participants viewed an infant-friendly video. The experimenters saw the gaze data visualised, and were guided as to positioning the participant relative to the eye tracker. Separate visual indicators showed the distance from the participant’s current position to the centre of the tracker box (where data quality is highest), in three dimensions. Once the experimenter was satisfied that the participant’s eyes were being tracked and their positioning was optimal, they moved to the calibration phase. Here a five-point calibration was automatically run, using infant-friendly stimuli (a colourful spiral which rotated as it shrank to engage attention). If enough valid data was collected after five calibration points, the results were plotted and presented to the experimenter, otherwise the five-point procedure was repeated. The experimenter judged the accuracy and precision of the calibration from the calibration plot, and chose to either proceed with the experimental tasks, or to re-run the five-point calibration.

Accuracy and precision were calculated during the gaze-contingent fixation stimulus that preceded each trial. The AOI around the fixation stimulus was 1.75x larger than the stimulus itself. The trial would begin even under conditions of high accuracy drift. Because the fixation stimulus was always at a known location, and because the trial would not begin until that location was fixated, we can use it to calculate the spatial error between the true gaze location and the gaze location reported by the eye tracker. Accuracy was calculated as the root-mean-square (RMS) of the euclidean distance between the location of each gaze sample and the location of the fixation stimulus. Precision was calculated as the RMS of the euclidean distance between each gaze sample and the centroid of all gaze samples.

## 2.2 Gap-overlap task

### 2.2.1 Stimulus presentation

The gap-overlap task [(Elsabbagh et al., 2009, 2013)](https://www.zotero.org/google-docs/?XYZsEm) measures the efficiency of shifts in attention from a central to a peripheral stimulus under competition and non-competition conditions. Trials were presented in blocks of 12. All stimuli were presented at a size of 3cm x 3cm (2.86° x 2.86° at 60cm viewing distance). Reward stimuli were either a star, a sun, a dog, cat, pig, tiger or tortoise which were animated and accompanied by a sound. Each trial started with the onset of a central stimulus (CS), a cartoon image of an analogue clock accompanied by an alerting sound. After a 200ms period had elapsed, the peripheral stimulus (PS) was presented. In the baseline condition the CS was removed from the screen when the PS was presented. In the overlap condition the CS continued to be presented for the duration of the rest of the trial. In the gap condition the CS was removed from the screen and the PS was presented after a short gap. The PS was a cartoon cloud that appeared on either the left or the right side of the screen and was accompanied by a sound, 3cm (2.86°) from the edge, rotating at 500° per second until fixated by the participant. A reward stimulus was then presented at the location of the PS for 1000ms.

Gap/overlap trials were presented in blocks of 12. Within these, four baseline, gap and overlap trials were presented, two of each on the left of the screen, and two on the right. The order of trials within a block was randomised, but with the constraints that no more than three trials in a row could be to the same side, or of the same condition. This same presentation order and logic was applied equally across all blocks and for all participants. Four blocks of twelve trials were initially presented, and in cases where online validation reported fewer than 12 valid trials per condition, an additional fifth block was presented.

### 2.2.2 Data extraction:

Mean saccadic reaction times (SRTs) were initially calculated for the Gap, Overlap and Baseline conditions. These were computed separately, using only valid trials. A trial was valid if the following conditions were met: 1) gaze fell on the CS; 2) no gaps of missing data longer than 200ms were present during the CS period (before PS onset); 3) there was at least one sample of gaze on the CS within 50ms either side of PS onset; 4) no gaps of missing data longer than 100ms were present during the PS period (between PS onset and reward onset); 5) SRT was longer than 150ms and shorter than 1200ms; 6) gaze did not go in the opposite direction to the side of the PS; 7) gaze did not enter the PS AOI after engagement with the CS but before PS onset. Participants with fewer than six valid trials per condition were removed from the analysis.

## 2.3 Face Pop-out task

### 2.3.1 Stimulus presentation:

The face pop-out task [(Gliga et al., 2009)](https://www.zotero.org/google-docs/?Kk9a9C) involves the infant viewing a series of slides containing a face, scrambled face, car, bird and phone while their gaze direction and duration is measured. Infants were presented with a series of six annular visual arrays each composed of five objects in different locations on the screen (Gliga et al., 2009; Hendry, Jones, Bedford, Gliga, Charman, Johnson, et al., 2018). Each array contained: 1) a face with direct gaze; 2) a visual ‘noise’ image generated from the same face presented within the array by randomising the phase spectra of the face whilst keeping the amplitude and colour spectra constant to act as a control for the low-level visual properties of the face stimuli (Halit et al., 2004); 3) a bird; 4) a car; and, 5) a mobile phone. Each array was presented for 10 seconds and counter-balanced for the location of the face in the array. The stimulus array was presented full-screen with adjustments for a proper aspect ratio, at 43.8cm x 28.6cm (39.0° x 26.8° @ 60cm). The convex hull of the popout array had a diameter of 26.8° at 60cm viewing distance. The individual elements of the array were not all of the same width and height due to differences in the underlying shape of the object they depicted (for example, the phones were taller and narrow than the cars). The longest dimension of each array was maintained between 7.8° and 9.6° (at 60cm) for all stimuli. The shortest dimension was calculated against the longest dimension to maintain a correct aspect ratio. For each element of the array, the AOI mask was formed by colouring each non-background pixel, then dilating the mask by 2° of visual angle.

### 2.3.2 Data extraction:

Areas-of-interest (AOIs) masks were placed around each stimulus, expanded by 2 degrees from the relevant picture to account for variation in data quality. Each sample of gaze was converted into a logical vector of “AOI scores”, marked as 1 when gaze fell inside the AOI for a particular sample, and 0 where it did not. We then “interpolated” (or, more accurately, filled in) samples in this vector where the gaze data was missing. To ensure that we do not erroneously assign missing data to one AOI when in fact gaze during the missing period moved to another AOI, we only do this for a) runs of missing data where the valid data on either side of the missing run was to the same AOI, and b) runs with a duration <200ms.

The proportion of samples within each AOI was calculated as number of samples in AOI / number of valid (non-missing) samples. Contiguous runs of samples within an AOI were identified and the mean proportion looking time and peak look duration to each AOI were calculated across valid trials only. Trials were marked as invalid if either a) the proportion of valid (non-missing) samples was less than 25%, or b) the duration of data was less than 5s. Data points were included in the analysis if the infant saw at least 3 Popout slides; proportion of lost data was not greater than 2 standard deviations from the sample mean; and the analysed values were not more than 2 standard deviations above or below the sample mean. Extracted variables were normed across all infants with valid data.

# Further details of Mullen Scales of Early Learning

The MSEL (Mullen, 1995) is a standardised measure that assesses developmental ability across five domains: Gross Motor, Visual Reception, Fine Motor, Receptive Language and Expressive Language. These five domains are then used to compute the MSEL Early Learning Composite (ELC) standard score. The MSEL (Mullen, 1995) was administered at all time points by trained researchers in the STAARS team. To allow for the greatest level of replicability and consistency across examiners, we have extremely strict guidelines about how Mullens should be administered and marked (see SMx.x). To this end, our guidelines for Mullen scoring include only behaviours that are captured on camera (so can be confirmed by a second/third researcher if necessary) within the Mullen session. For example, if an infant demonstrates babbling throughout the rest of the testing day (i.e., during another task or a lunch break), but not during the specific Mullen administration session, we would not score this infant as being able to produce babbling sounds on the Expressive Language scale. To further ensure the fidelity of the scoring, a second fully trained researcher watches the administration in real time (via a video feed) and consensus discussions take place after the testing session. These strict administration and scoring guidelines (although those recommended in the Mullen manual) may not be those applied more broadly in the field, and thus may account for relatively poorer performance in this cohort at infant timepoints relative to US norms. We present standard scores for overall developmental level for descriptive cohort comparisons (see Table x).

# Results

## 4.1 SM Analysis 1: Data quality

We first compared data quality metrics between the NF1 and TD groups.

*Accuracy*

The model with a random slope for age would not converge; the final model was of the form (scores ~ Age * group+sex, random = list(~1| ID; AIC=206.26, BIC=226.06, log likelihood = -96.13). There were no significant differences in accuracy between the NF1 and control groups (t(55)=-1.1, p = 0.28); accuracy improved with age (t(70)=-4.48, p < 0.001) but this did not vary by group ( t(70)=1.1, p = 0.28). Female infants had slightly greater accuracy than male infants (t(55)=-2.59, p = 0.01).

*Precision*

The final model was of the form (scores ~ Age * group+sex, random = list(~Age| ID; AIC=282.92, BIC=308.37, log likelihood = -132.46). Precision values were significantly lower in the NF1 group, indicating better precision (t(55)=-4.89, p <0.001); improved with age (t(70)=-5.39, p < 0.001) and the change was steeper in the controls ( t(70)=2.82, p = 0.006). Female infants had slightly higher precision values (worse precision) than male infants (t(55)=2.81, p = 0.006). Precision was thus included as a covariate in sensitivity analyses for all relevant sections.

*Gap (trials per condition)*

Average trials per condition are shown in Table S1. The model with a random slope for age would not converge; the final model was of the form (scores ~ Ageindays * group+sex, random = list(~1| condition,~1| ID); AIC =1989.98; BIC=2021.25; log likelihood=-986.99). There was a marginally significant main effect of age (t(200)=1.82, p = 0.07), no significant group differences (t(166)=-0.59, p = 0.56) or sex differences ((t(166)=-0.84, p = 0.40) or interactions between group and age (t(200)= 0.62, p = 0.53).

| Table S1: Mean number of valid trials in the Gap eye tracking task | | | | | |
| --- | --- | --- | --- | --- | --- |
| 5 months |  |  |  |  |  |
|  | LL | NF1 | ASD | ADHD | ASD+ADHD |
| Gap Baseline | 13.68 (4.44) | 13.82 (3.89) | 13.87 (5.7) | 15.4 (6.07) | 13 (4.9) |
| Gap Gap | 13.08 (4.08) | 13.45 (3.91) | 13.02 (5.3) | 14.8 (5.32) | 11.93 (4.91) |
| Gap Overlap | 12.08 (4.32) | 13.36 (3.56) | 13.43 (5.21) | 14.2 (5.17) | 11.86 (4.85) |
|  |  |  |  |  |  |
| 10 months |  |  |  |  |  |
|  | LL | NF1 | ASD | ADHD | ASD+ADHD |
| Gap Baseline | 16.12 (2.49) | 14.42 (5) | 13.93 (4.48) | 15.81 (4.6) | 14.24 (3.65) |
| Gap Gap | 15.54 (3.37) | 13.79 (4.72) | 13.49 (4.25) | 14.98 (4.78) | 14.43 (3.83) |
| Gap Overlap | 15.77 (3.45) | 14.11 (4.82) | 13.47 (4.77) | 14.54 (4.33) | 14.67 (3.55) |
|  |  |  |  |  |  |
| 14 months |  |  |  |  |  |
|  | LL | NF1 | ASD | ADHD | ASD+ADHD |
| Gap Baseline | 15 (3.1) | 16 (2.7) | 14.39 (4.19) | 14.75 (5.41) | 15.17 (2.5) |
| Gap Gap | 13.48 (3.23) | 15.22 (2.89) | 13.85 (3.78) | 14.83 (5.14) | 14.17 (2.92) |
| Gap Overlap | 14.87 (2.79) | 14.74 (3.33) | 13.85 (3.84) | 14.83 (5.75) | 14.44 (2.23) |

*Popout: (Mean proportion of valid samples )*

The final model was of the form (proportion valid samples ~ Age * group+sex, random = ~Age| ID; AIC= 377.44, BIC= 402.30, log likelihood=-179.72). There were no group (t(52)=0.12, p = 0.91) or sex (t(52)=1.12, p = 0.26) differences in the proportion of valid samples and no interaction with age (t(55)=-0.25, p = 0.81) or main effect of age (t(65)=-0.10, p = 0.91).

## 4.2 SM Analysis 2: Confirmation of task performance

First, we confirmed that the cohort as a whole showed the expected profile of performance on the tasks. To do this we used the non-transformed data in the whole cohort (NF1, Typical Likelihood, infants with a family history of ASD and/or ADHD).

*Gap*

The final model was of the form scores ~ Ageindays * condition, random = list(~Ageindays| ID; Model AIC=-1421.2, BIC = -1370.1, LL = 720.6). This confirmed that reaction times were faster in the Gap than the baseline condition (t(1047)=-5.44, p < 0.001) and longer in the overlap condition than the Baseline condition (t(1047)=16.95, p < 0.001). Reaction times decreased with age (t(1047)=-5.29, p < 0.001); this varied by condition such that the slope was steeper in the Gap condition (t(1047)=-3.81, p = 0.001) and the Overlap condition (t(1047)=-7.60, p < 0.001), confirming developmental improvements in the facilitation and disengagement effects.


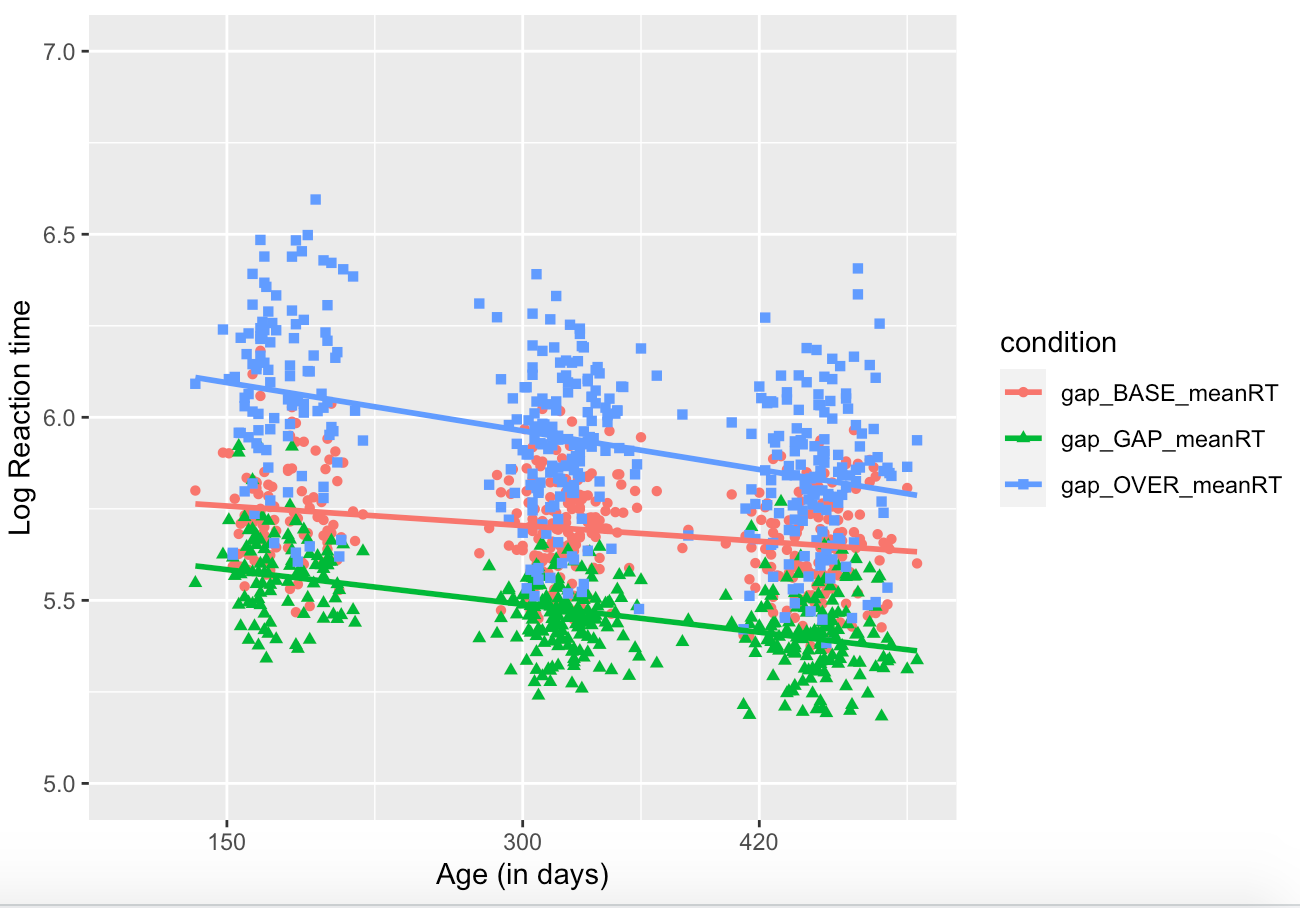


*Popout*

*First look*: Infants were more likely to orient to the face than expected by chance (0.2) in the pop-out task (M = 0.57, SD =0.22; t(203)=24.7, p < 0.001). The model was of the form scores ~ Ageindays, random = list(~Ageindays| ID; Model AIC= -23.59751, BIC= -3.747906, LL= 17.79876. There was no change with age (t(57) = =1.32, p = 0.19).

*Proportion looking*:

The model was of the fomr scores ~ Ageindays * condition, random = list(~Ageindays| ID; Model AIC = -3486.298 , BIC = -3408.64 LL = 1757.149). Infants spent more time looking at the face than all other areas of interest (ts(1717) = (car)-20.23,(phone) -23.36, (bird)-24.48, (noise)-22.48; ps < 0.001).


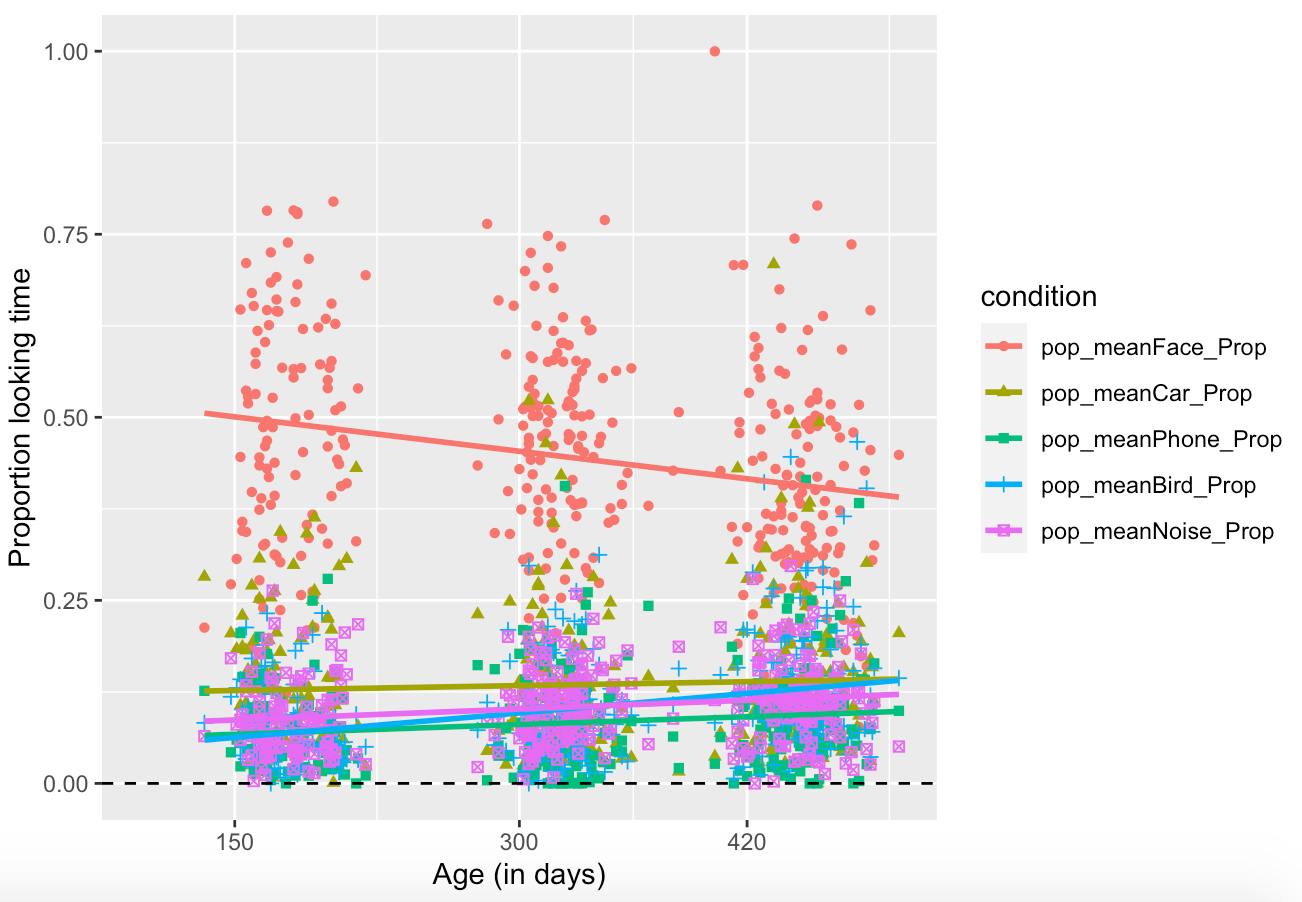


Disengagement and facilitation

For disengagement, sex was not retained in the model as it was not a significant predictor (t(54) = 0.38, p =0.71). Thus, model 1 was selected as the final model (Model1: AIC=230.9, BIC = 253.32, loglik = -107.44). There were no differences between the NF1 and all other groups in disengagement (t(55) = 0.76, p =0.45) and no group differences in change with age (t(67) = 0.81, p =0.42).

For facilitation, sex was not retained in the model as it was not a significant predictor (t(54) = 0.89, p =0.37). Thus, the final model was m1<-lme(scores ~ Ageindays * label, random = ~Ageindays| ID, data=newdata; AIC=357.48, BIC = 379.91, loglik = -170.73). There were no differences in facilitation time between the NF1 and all other groups (t(55) = 0.09, p =0.92) and no group differences in change with age (t(67) = 0.16, p =0.87).

## 4.3: SM Analysis 3: Controlling for precision

We included metrics of eyetracking accuracy and precision in our models examining differences between infants with NF1 and those with typical development

*Exogenous shifting*

Gap reaction times: If precision was included in the model, there was a main effect of precision such that higher precision associated with slower reaction times (t(199)=-2.0, p = 0.047) but other effects remained the same (effect of group t(166)=-3.29, p = 0.0012; effect of group by age t(199)=2.65, p = 0.0087; effcct of age t(199)=-8.69, p < 0.001).

*Endogenous attention*

First looks to face

Controlling for precision rendered these differences weaker (NF1 orienting to face relative to typical infants t(51)=-1.78, p = 0.08; change with age in NF1 relative to controls t(51)=1.71, p = 0.09; effect of sex t(51)=1.26, p = 0.21).

Proportion looking to face

Controlling for precision rendered similar results (face looking decreased with age (t(51)=-3.13, p = 0.003; effect of age was smaller in NF1 (t(51)=2.34, p = 0.02; no overall group difference t(52)=-1.65, p = 0.1).
